# Berberine Derivative Compound 13 as a Potent Promoter of Osteoblast Differentiation via Akt and PKC Signaling Pathways

**DOI:** 10.3390/ijms26072984

**Published:** 2025-03-25

**Authors:** Meiyu Piao, Youn Ho Han, Kwang Youl Lee

**Affiliations:** 1 Research Institute of Pharmaceutical Sciences, College of Pharmacy, Chonnam National University, Gwangju 61186, Republic of Korea; my101park@gmail.com; 2 Department of Oral Pharmacology, College of Dentistry, Wonkwang University, Iksan 54538, Republic of Korea

**Keywords:** berberine, compound **13**, osteoblast differentiation, Akt, PKC

## Abstract

Berberine has been widely studied for its biological functions in various diseases, including cancer, diabetes, and cardiovascular diseases. Nevertheless, structural modifications of berberine have been demonstrated to augment its pharmacological efficacy in specific biological processes, particularly osteogenesis. In this study, we aimed to explore new berberine derivatives with pro-osteogenic activity and molecular mechanisms. Our results demonstrated that compound **13** is the most effective among the tested compounds. Compound **13** significantly enhanced BMP4-induced alkaline phosphatase (ALP) staining and increased the transcriptional activity of osteogenic markers such as ALP, Runt-related gene 2 (Runx2), and Osterix at both the mRNA and protein levels. Furthermore, we found that the Akt and PKC signaling pathways play crucial roles in compound **13**-induced osteogenesis via treatment with specific inhibitors. The molecular docking results supported the potential interaction between compound **13** and these kinases. These findings highlighted the regulatory role of compound **13** in osteoblast differentiation via the Akt and PKC signaling pathways. Overall, our study provides compelling evidence that compound **13** is a promising therapeutic candidate for the treatment of osteoporosis, with the potential for further development and optimization to improve bone health and strength.

## 1. Introduction

Osteoporosis is a clinical skeletal disorder characterized by reduced bone strength and an increased tendency for fracture [1]. Bone density and mass are the key parameters affecting bone strength. Many factors influence these two parameters, including age, postmenopausal hormonal changes, and family history [2]. Bone remodeling, which includes both bone formation and resorption, plays a crucial role in the pathogenesis of osteoporosis [3]. Maintaining the dynamic balance between osteoblasts and osteoclasts is the primary treatment strategy for osteoporosis. Although several drugs targeting bone resorption have been identified, their long-term uses are limited by severe side effects [4]. Therefore, developing novel therapeutic agents with improved efficacy and safety is urgently needed.

Osteoblasts are the primary cell types responsible for bone formation [5]. Various cytokines, growth factors, and hormones are involved in bone formation by regulating osteoblast proliferation and differentiation [6]. Also, there are several signaling pathways and transcription factors that play major roles in controlling osteoblast differentiation. These include bone morphogenetic proteins (BMP), transforming growth factor-β (TGF-β), Wnt, the Hedgehog signaling pathway, and the osteoblast differentiation-specific transcription factors Runt-related gene 2 (Runx2) and Osterix (Osx) [7,8,9]. For instance, BMP4 induces osteoblast differentiation via both Smad4 and mTORC1 signaling [10]. Furthermore, mice lacking either Runx2 or Osx show a complete lack of intramembranous and endochondral ossification [11]. This evidence suggests an important role for signal transduction and transcriptional regulation in osteoblast differentiation.

**Figure 1:**
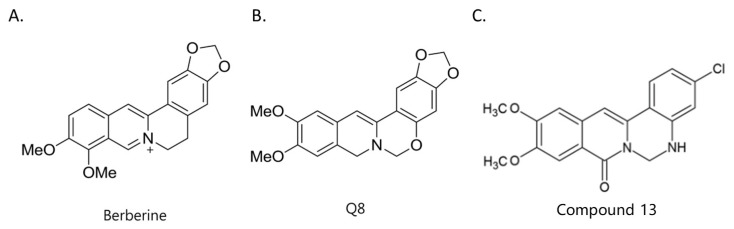
Structures of (**A**) berberine, (**B**) Q8, and (**C**) compound **13**.

Berberine, an isoquinoline alkaloid, has recently been studied to elucidate its diverse pharmacological and therapeutic effects, such as cancer and inflammation [12,13,14]. In osteoblast differentiation, berberine facilitates osteogenesis by increasing Runx2 activity and activating the p38 signaling pathway [15]. Also, berberine promotes the osteogenic differentiation of bone marrow-derived mesenchymal stem cells via the canonical Wnt/β-catenin-signaling pathway [16]. Given the multi-function ability of berberine, an increasing number of studies have focused on the structural modification of berberine to maximize its effectiveness and pharmacological activity, leading to a series of berberine derivatives. For example, Q8 is one of these berberine derivatives, and it has been proven to have a positive role in osteogenic differentiation by inhibiting PPARγ [17]. In the present study, we demonstrated the effect of berberine derivative compound **13** during osteoblast differentiation (Figure 1).

## 2. Results

### 2.1. Structure–Activity Relationship of Berberine Derivatives Evaluated Through ALP Staining Assay

**Figure 2:**
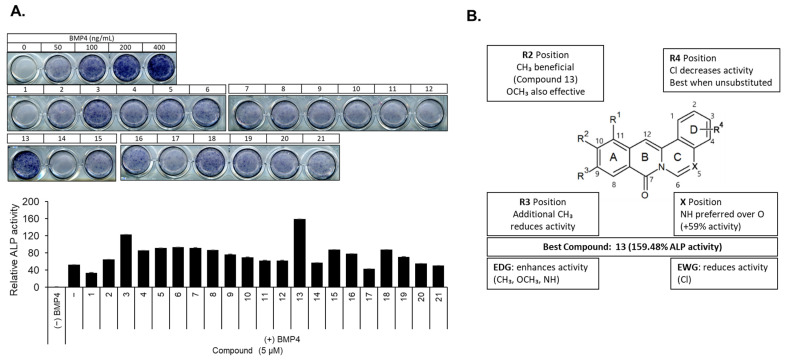
Compound **13** showed the most effective pro-osteoblastic activity. (**A**) C2C12 cells were induced by BMP4 and treated with the indicated berberine compound (5 μM) for 72 h, and the osteoblastic activity was analyzed by ALP staining. (**B**) Structure–activity relationship (SAR) of berberine derivatives for osteoblast differentiation. The schematic representation illustrates the key structural features influencing the osteogenic activity of the berberine derivatives. Electron donating group (EDG); electron withdrawing group (EWG).

Structure–activity relationship (SAR) analysis of the provided data highlighted how the nature and position of the substituents significantly influenced ALP activity across the tested compounds (Table 1, Figure 2A). A notable trend was observed with the substitution at R_3_; when OCH_3_ was present, as in compound **13**, it led to the highest ALP activity (159.48%), indicating that this specific group played a crucial role in enhancing the interaction with the enzyme. In contrast, the introduction of chlorine (Cl) at R_2_, R_3_, and R_4_ resulted in a substantial reduction in activity, as evidenced by the lower performance of compounds **14**–**21**. This suggests that chlorine may disrupt favorable interactions or alter the electronic or steric properties of a compound in a manner that hampers enzyme activation. Furthermore, the nature of the substituent at position X emerged as a critical factor, with compounds containing NH demonstrating consistently higher activity than those with O. For instance, compound **13** with NH significantly outperformed compounds **3** and **7**, which had O as the substituent at the same position, underscoring the importance of hydrogen bonding or other favorable interactions enabled by NH. The substitution patterns across the R groups also play a pivotal role. The number and type of substituents were directly correlated with the ALP activity, where combinations of methyl or methoxy groups, as observed in compound **13**, resulted in enhanced activity. These groups likely contribute to the optimal balance between hydrophobicity and electronic effects, facilitating stronger or more stable interactions with the active sites of the enzyme. Conversely, excessive or unfavorable substituents, such as multiple Cl groups, diminished the activity, suggesting steric hindrance or adverse electronic interactions. Compound **13** uniquely balanced these factors, featuring a CH_3_ group at R_2_ and NH at X, which collectively enhanced the ALP activity without introducing detrimental substituents that could negatively affect enzyme function. Compound **13** was selected as the lead compound because of its outstanding performance and structural attributes. It demonstrated the highest ALP activity among all tested compounds, indicating that its structural features are particularly well suited for optimal enzyme activation. Additionally, the substitution pattern strikes a balance between simplicity and effectiveness by avoiding the inclusion of chlorine or other substituents known to reduce activity. This simplicity also makes compound **13** an excellent candidate for further chemical modification and optimization, providing a robust foundation for the development of more potent derivatives. Additionally, electron donating groups (EDGs) like methyl (CH_3_) and methoxy (OCH_3_) generally enhanced the ALP activity, with compound **13** showing the highest activity (159.48%) due to its CH_3_ group at the R_2_ position. Conversely, electron withdrawing groups (EWGs) such as chlorine consistently reduced activity across compounds **14**–**21**. The NH group at position X in compound **13** (acting as an EDG through resonance) outperformed the more electronegative O group in similar structures. Optimal ALP activity appears to require a balanced electronic distribution with moderate electron donation, which explains compound **13**’s superior efficacy with its specific EDG arrangement (Figure 2B). Overall, compound **13** stands out not only for its superior activity but also for its versatility and potential for enhancement, making it the ideal choice for further investigation and application in enzyme-targeted studies.

### 2.2. Compound 13 Promotes Osteoblast Differentiation in C2C12 Cell

**Figure 3:**
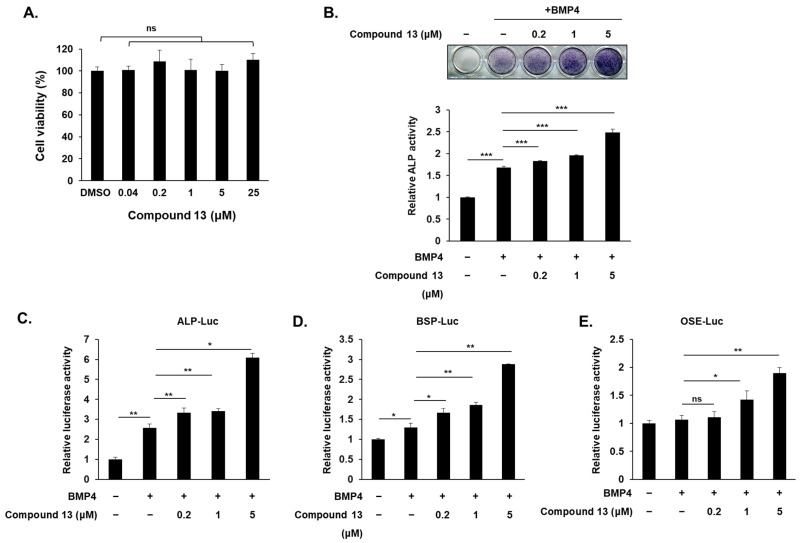
Compound **13** promotes osteoblast differentiation in C2C12 cells. (**A**) Cell viability of C2C12 cells treated with a series of compound **13** concentrations (0.04 μM to 25 μM) for 72 h. (**B**) Representative ALP staining and quantification of C2C12 cells after treatment with BMP4 and increasing concentrations of compound **13**. (**C**–**E**) C2C12 cells were transfected with ALP−Luc, BSP-Luc, and OSE-Luc reporter, respectively. After 24 h, the transfected cells were incubated with or without BMP4 alone or the BMP4 + compound **13** (0.2, 1, and 5 μM) for an additional 24 h. β-gal was used to normalize the transfection efficiency. The luciferase reporter activities were measured following 48 h transfection. Data are expressed as mean ± standard deviation (SD) (*n* = 3). ns: not significant, * *p* < 0.05, ** *p* < 0.01, *** *p* < 0.001.

Next, we examined the possible cytotoxic effects of compound **13** on the C2C12 cells; an MTT assay was used to analyze the cells. As shown in Figure 3A, no significant difference was observed between the compound **13** treatment and the untreated control. Therefore, all tested concentrations were suitable for future studies. To investigate the effects of compound **13** in osteoblast differentiation, we treated 0.2, 1, and 5 μM of compound **13** in BMP4-induced C2C12 cells for 72 h and performed ALP staining. ALP staining and quantitative analysis showed that compound **13** significantly enhanced BMP4-induced osteoblast differentiation in a dose-dependent manner (Figure 3B). Next, to elucidate whether compound **13** regulates the transcriptional activity of osteogenic genes, we examined the promoter activity of ALP, the bone sialoprotein (BSP)-promoter region, and osteoblast-specific *cis*-acting element 2 (OSE2). These were co-transfected with the reporter genes (ALP-Luc, BSP-Luc, and OSE-Luc) and β-galactosidase into C2C12 cells and treated with BMP4 and increasing concentrations of compound **13** for 48 h. Compound **13** further increased the BMP4-induced ALP, BSP, and OSE luciferase activities in a dose-dependent manner (Figure 3C–E). These results indicate that berberine derivative **13** plays a positive role in osteoblast differentiation.

### 2.3. Compound 13 Increases Osteogenic Gene Expression at mRNA and Protein Levels

**Figure 4:**
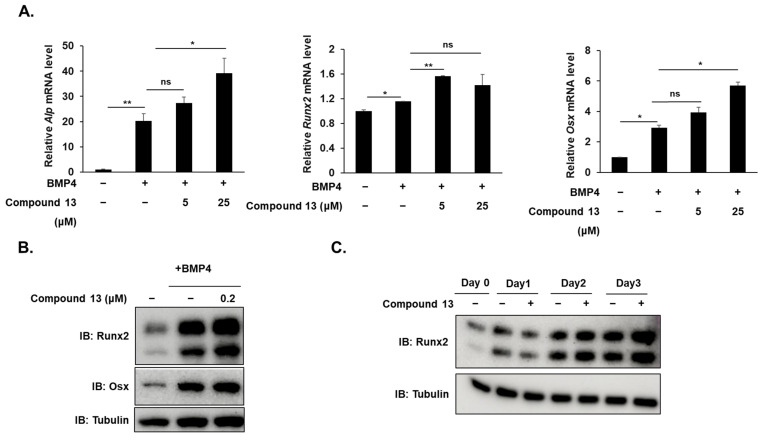
Compound **13** increased osteogenic gene expression at mRNA and protein levels**.** (**A**,**B**) C2C12 cells were induced by BMP4 and treated with or without compound **13** for 72 h. (**A**) mRNA levels of *Alp*, *Runx2*, and *Osx* were analyzed by RT-qPCR. *Gapdh* was used as a loading control. Data are expressed as mean ± standard deviation (SD) (*n* = 3). ns: not significant, * *p* < 0.05, ** *p* < 0.01. (**B**) Protein levels of Runx2 and Osx were detected by immunoblotting. Tubulin was used as a loading control. (**C**) C2C12 cells were incubated with or without compound **13** and harvested by indicated time points (days 0, 1, 2, and 3). The protein level of Runx2 was detected by immunoblotting. Tubulin was used as a loading control.

To further elucidate the positive role of compound **13**, we examined the mRNA and protein levels of osteogenic genes, such as ALP, Runx2, and Osx. BMP4 induces osteoblast differentiation and increases the mRNA levels of *Alp*, *Runx2*, and *Osx*. Compound **13** further enhanced BMP4-induced mRNA expression (Figure 4A). In addition, when using a low concentration (0.2 μM) of compound **13**-treated BMP4-induced C2C12 cells for 48 h, compound **13** markedly increased the protein expression of Runx2 and Osx (Figure 4B). Furthermore, compound **13** further increased BMP4-induced Runx2 protein expression during osteogenesis, as compared to the untreated group, and the protein level of Runx2 was highest on day 3 (Figure 4C). These results are consistent with the hypothesis that compound **13** promotes osteoblast differentiation.

### 2.4. Akt and PKC Signaling Involved in Compound 13-Regulated Osteoblast Differentiation

**Figure 5:**
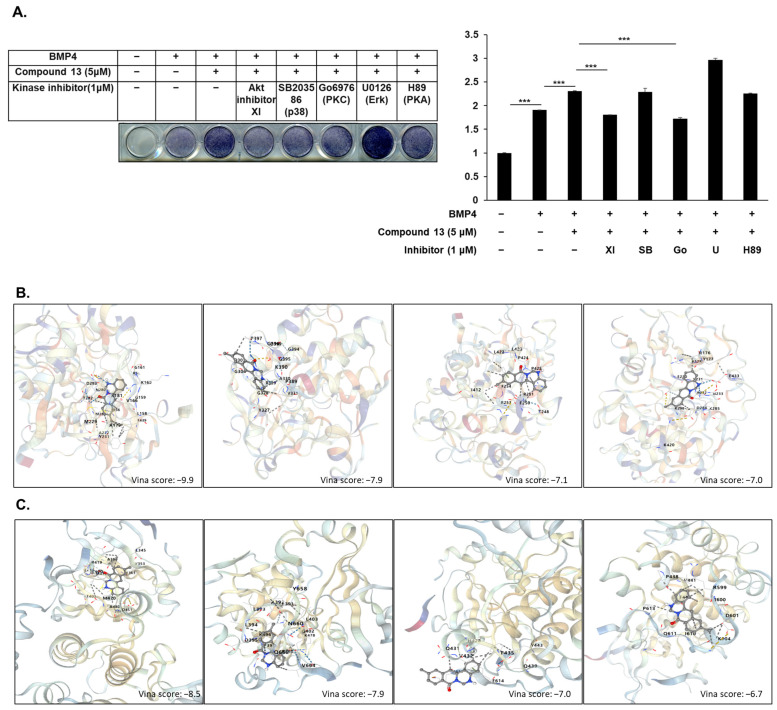
Akt and PKC signaling involved in compound **13** regulated osteoblast differentiation. (**A**) C2C12 cells were induced by BMP4 and treated with compound **13** (5 μM) and a series of kinase inhibitors (1 μM) for 72 h, and were analyzed for osteoblastic activity by ALP staining. Data are expressed as mean ± standard deviation (SD) (*n* = 3). *** *p* < 0.001. (**B**,**C**) Docking models of compound **13** Akt and the PKC protein. Three-dimensional solid ribbon indicates the structure of the Akt (**B**) and PKC (**C**), respectively, and the compound 3b (various color)-binding active site of Akt or PKC is indicated as a dotted line. The Vina Score represents the estimated binding free energy (ΔG) between the ligand and protein, with lower scores indicating stronger binding affinity.

Osteoblast differentiation is a complex and diverse process regulated by multiple signaling pathways and transcription factors [8,18]. To elucidate the mechanism by which compound **13** regulates osteoblast differentiation, we treated compound **13** and a series of kinase inhibitors in BMP4-induced C2C12 cells. We treated the cells with an Akt signaling pathway inhibitor (XI), a p38 signaling pathway inhibitor (SB203586), a PKC signaling pathway inhibitor (Go6976), an ERK signaling pathway inhibitor (U0126), or a PKA signaling pathway inhibitor (H89) at the endpoint of differentiation before 24 h. ALP staining showed that the Akt signaling inhibitor (XI) and the PKC signaling pathway inhibitor (Go6976) dramatically decreased compound **13**-enhanced osteoblast differentiation (Figure 5A). We further explored the potential molecular interactions between compound **13** and the signaling proteins Akt and PKC using molecular docking analysis. A docking study was conducted to predict and visualize the binding modes of compound **13** within the active sites of Akt and PKC, using crystal structures obtained from the Protein Data Bank (PDB: Akt-3O96; PKC-4RA4). The analysis revealed that compound **13** formed stable interactions with critical residues in the catalytic domains of both Akt and PKC, suggesting a strong binding affinity and specificity for these kinases (Figure 5B,C). These results indicate that Akt and PKC signaling are required for compound **13**-regulated osteoblast differentiation.

## 3. Discussion

In this study, we identified and characterized a novel berberine derivative, compound **13**, which demonstrated significant pro-osteogenic activity during osteoblast differentiation. Through a comprehensive analysis, we showed that compound **13** enhanced BMP4-induced osteoblast differentiation by upregulating osteogenic markers such as Alp, Runx2, and Osterix at both transcriptional and protein levels. Importantly, our findings revealed that the Akt and PKC signaling pathways play essential roles in mediating the osteogenic effects of compound **13**. These results not only support the growing evidence of the pharmacological potential of berberine derivatives but also shed light on the molecular mechanisms underlying their osteogenic activities.

The pharmacological value of berberine has been demonstrated in various diseases, including cancer [19], diabetes [20], and cardiovascular disease [21]. In addition, the structural modifications of berberine are more effective and have better prospects [22]. For example, berberine promotes osteoblast differentiation [15]. Berberine bioisostere Q8 showed the most effective activity as compared to berberine, regulating both Runx2 and Osx transcriptional activity [23]. In this context, compound **13** emerged as a potent new derivative with superior activity, demonstrating the highest osteoblastogenic potential among the compounds tested. Unlike its predecessors, compound **13** enhanced osteogenic marker expression while maintaining a simple and effective structural framework, making it an attractive candidate for further development.

To further elucidate the mechanism of action of compound **13** in osteoblast differentiation, we examined the effects of kinase inhibitors on compound **13**-regulated osteogenesis. We found that the Akt and PKC signaling inhibitors, Go6976 and XI, strongly suppressed the effects of compound **13**. According to the docking results, Akt and PKC interacted with compound **13**. The regulatory impact of Akt and PKC signaling on osteoblast differentiation has been fully studied in previous research. The PKC pathway plays a central role in fibroblast growth factor-stimulated expression and the transactivation of Runx2 [24]. Moreover, PKC signaling is required for fibroblast growth factor receptor 2-regulated osteoblast differentiation [25]. Akt, also known as protein kinase B (PKB), is essential for BMP2-mediated osteoblast differentiation and bone development [26]. Furthermore, Akt regulates the transcriptional activities of both Runx2 and Osx by enhancing protein stability [27,28]. Our findings align with these established roles and highlight the dual involvement of Akt and PKC in mediating the osteogenic effects of compound **13**. In a docking study, compound **13** was found to engage in hydrogen bonding with key residues within the ATP-binding pocket, along with hydrophobic interactions that stabilized its binding. These interactions likely enhanced the ability of the compound to modulate Akt activity, either by facilitating its activation or by stabilizing its active conformation during osteoblast differentiation. Similarly, compound **13** demonstrated a complementary fit within the kinase domain, establishing hydrogen bonds and van der Waals interactions with residues essential for PKC enzymatic function. These findings implied that compound **13** may act as a direct modulator of PKC activity, potentially enhancing its downstream signaling effects. However, further studies are necessary to confirm these interactions and elucidate additional downstream effects. Compound **13** has significant potential as a therapeutic candidate for osteoporosis. Osteoporosis is a debilitating skeletal disorder characterized by imbalanced bone remodeling, in which bone resorption outweighs bone formation. Current treatments targeting bone resorption are associated with severe side effects that limit their long-term uses. Therefore, the ability of compound **13** to enhance osteoblast differentiation and promote osteogenesis makes it a promising alternative. By targeting key transcription factors and signaling pathways, compound **13** may help restore the dynamic balance between osteoblast and osteoclast activities, thereby addressing the underlying pathophysiology of osteoporosis. Furthermore, its high efficacy and considerable safety render it a promising candidate for clinical development.

## 4. Materials and Methods

### 4.1. Reagent and Antibody

Berberine derivatives, including compound **13**, were synthesized as previously described [29]. The MAPK inhibitor (U0126), PKA inhibitor (H89), PKC inhibitor (Go6976), p38 MAPK inhibitor (SB203580), and Akt inhibitor (XI) were purchased from Calbiochem (San Diego, CA, USA). The Runx2 (sc-390351; 1:1000), Osx (sc-393325; 1:1000), and α-tubulin (sc-8035; 1:1000) antibodies were purchased from Santa Cruz Biotechnology (Santa Cruz, CA, USA). The anti-Myc (9E10; 1:1000) and anti-HA (12CA5; 1:1000) antibodies were purchased from Roche Applied Science (Basel, Switzerland).

### 4.2. Cell Culture and Differentiation

C2C12 cells were purchased from ATCC (American Type Culture Collection, Manassas, VA, USA). C2C12 cells were maintained in Dulbecco’s Modified Eagle Medium (DMEM) (#12100046; Gibco™, Carlsbad, CA, USA), supplemented with 10% fetal bovine serum (FBS) (S001-07; Welgene Inc, Deagu, Republic of Korea) and 1% antibiotic–antimycotics (#15240062; Gibco™) and cultured in an incubator containing 5% CO_2_ at 37 °C, and cells were passaged every 2 days. For osteoblast differentiation [30], the C2C12 cells were cultured in DMEM supplemented with 2% FBS and bone morphogenetic protein 4 (BMP4) (50 ng/mL).

### 4.3. MTT Assay

3-(4,5-dimethylthiazol-2-yl)-2,5-diphenyl tetrazolium bromide (MTT; Amrescom, Solon, OH, USA) was used to examine cell viability [31]. The C2C12 cells were seeded into 96-well plates and incubated with or without compound **13** for 72 h. After incubation, the MTT solution was added to each well and incubated for 4 h. Next, the MTT solution was removed, and the MTT formazan crystals were dissolved in isopropanol. Absorbance was measured at 510 nm using a microplate reader.

### 4.4. Alkaline Phosphatase (ALP) Staining

C2C12 cells were treated with or without compound **13** and incubated in the differentiation medium for 72 h. After 72 h of differentiation, the cells were washed twice with phosphate-buffered saline (PBS) and fixed with 4% paraformaldehyde (PF) at room temperature for 10 min. Fixed cells were washed twice with PBS, then 0.3 mL of 1-Step™ NBT/BCIP Substrate Solution (34042; Thermo Scientific, Waltham, MA, USA) was added for 15 min at room temperature in the dark. ALP staining was quantified by measuring absorbance at 480 nm [32].

### 4.5. Luciferase Assay and Transfection

C2C12 cells were transfected with 0.2 μg of luciferase report genes, such as ALP-Luc, BSP-Luc, and OSE-Luc, by using the polyethyleneimine (PEI) transient transfection method (Polysciences, Inc., Warrington, PA, USA) [33]. After 24 h of transfection, the cells were transferred to differentiation media containing BMP4 and treated with increasing concentrations of compound **13** for 48 h. To measure luciferase activity, the samples were extracted using the Cell Culture Lysis Reagent (Cat.# E1531, Promega, Madison, WI, USA) and a luciferase reporter gene test kit (E1501; Promega) following the manufacturer’s instructions. All experiments were performed in triplicates.

### 4.6. Reverse Transcription Followed by Quantitative Polymerase Chain Reaction (RT-qPCR)

C2C12 cells were incubated with BMP4 and increasing concentrations of compound **13** for 3 d, and the total RNA was isolated using TRIzol reagent (TaKaRa, Tokyo, Japan) according to the manufacturer’s protocol. Approximately, 1 μg of RNA was converted to cDNA with oligo (dT) primers and GoScript™ Reverse Transcriptase (A5001; Promega) following the manufacturer’s instructions. The synthesized cDNA was analyzed by RT-qPCR, using a SYBR Premix Ex Taq kit (RR420A; TaKaRa) [34]. The primer sequences for PCR were as follows: m*Alp* forward 5′-ATC TTT GGT CTG GCT CCC ATG-3′ and reverse 5′-TTT CCC GTT CAC CGT CCA C-3′; m*Runx2* forward 5′-CCT GAA CTC TGC ACC AAG TCC T-3’ and reverse 5′-TCA TCT GGC TCA GAT AGG G-3′; m*Osx* forward 5′-TCG CAT CTG AAA GCC CAC TT-3’ and reverse 5′-CTC AAG TGG TCG CTT CTG GT-3′; and m*Gapdh* forward 5′-AGG TCG GTG TGA ACG GAT TTG-3’ and reverse 5′-GGG GTC GTT GAT GGC AAC A-3′.

### 4.7. Immunoblotting

The whole-cell lysates were extracted using ice-cold lysis buffer (25 mM HEPES (pH 7.4), 150 mM NaCl, 1% NP-40, 0.25% sodium deoxycholate (Na-Doc), 10% Glycerol, 25 mM NaF, 1 mM EDTA, 1 mM Na_3_VO_4_, 250 μM PMSF, 10 μg/mL leupeptin, 10 μg/mL aprotinin, and 10 μg/mL peptidase). The cellular lysates were cleared by centrifugation at 13,200 rpm at 4 °C, and the supernatants were subjected to immunoblotting. All protein samples were resolved using SDS-PAGE and transferred to polyvinylidene fluoride membranes (PVDF, Immobilon-P; Millipore, Burlington, MA, USA) [35]. The membranes were blocked with 5% skim milk and incubated with appropriate primary antibodies, followed by incubation with horseradish peroxidase (HRP)-conjugated secondary antibodies. Subsequently, antibody-specific proteins were visualized using Immobilon Western Chemiluminescent HRP Substrate (WBKLS0500; Millipore). Protein bands were detected and analyzed using the Amersham^TM^ ImageQuant^TM^ 800 system (GE Healthcare Life Sciences, Marlborough, MA, USA).

### 4.8. Molecular Docking

Molecular docking of the synthesized berberine-derivative compound **13** with Akt or PKC was performed using a cavity detection-guided blind docking program (Yang Cao Lab, Chengdu, China). The protein structures of Akt (PDB: 3O96) and PKC (PDB: 4RA4) were prepared by removing water molecules and adding hydrogen atoms. The energy-minimized structure of compound **13** was systematically docked using a two-step approach: automatic identification of potential binding cavities followed by unbiased docking simulations at each detected site. Multiple conformations were generated and evaluated based on a scoring function accounting for hydrogen bonding, van der Waals interactions, and desolvation effects, with focus on the interactions with catalytically important residues in both kinases. The Vina Score is directly related to binding energy, as it represents an estimated binding free energy (ΔG) between a ligand and a protein, measured in kcal/mol. Lower Vina Scores indicate stronger binding affinity, meaning that the ligand–protein interaction is more stable. The score incorporates various interactions, including van der Waals forces, hydrogen bonding, electrostatic interactions, and desolvation effects. Because Vina Score is a negative value, a more negative score signifies a more favorable binding interaction.

### 4.9. Statistical Analysis

All experimental results were analyzed using the Student’s *t*-test, and all experiments were performed in triplicate and repeated at least three times. Statistical significance was set at *p* < 0.05.

## 5. Conclusions

In summary, our study demonstrated that compound **13**, a novel berberine derivative, promoted osteoblast differentiation through the Akt and PKC signaling pathways. These findings provide a strong foundation for the further exploration and optimization of berberine derivatives as therapeutic agents for bone-related disorders. Future studies should focus on detailed mechanistic evaluations, in vivo efficacy, and the potential of compound **13** for integration into clinical applications in the treatment of osteoporosis and other conditions affecting bone health.

## Figures and Tables

**Table 1:** Structure and activity relationship of berberine derivatives.

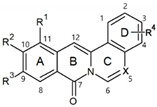							
No.	Structure	R_1_	R_2_	R_3_	R_4_	X	ALP Staining (%)
**1**	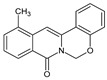	CH_3_-	-	-	-	O	33.80
**2**	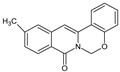	-	CH_3_	-	-	O	64.91
**3**	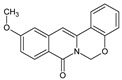	-	OCH_3_	-	-	O	123.23
**4**	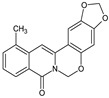	CH_3_	-	-	OCH_2_O	O	85.83
**5**	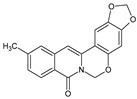	-	CH_3_	-	OCH_2_O	O	91.39
**6**	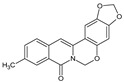	-	-	CH_3_	OCH_2_O	O	93.70
**7**	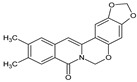	-	CH_3_	CH_3_	OCH_2_O	O	92.08
**8**	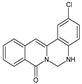	-	-	-	Cl	NH	86.66
**9**	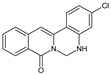	-	-	-	Cl	NH	76.60
**10**	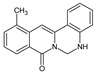	CH_3_	-	-		NH	69.93
**11**	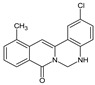	CH_3_	-	-	Cl	NH	62.58
**12**	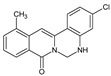	CH_3_	-	-	Cl	NH	62.31
**13**	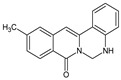	-	CH_3_	-	-	NH	159.48
**14**	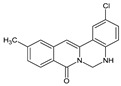	-	CH_3_	-	Cl	NH	57.25
**15**	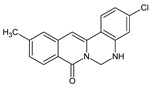	-	CH_3_	-	Cl	NH	87.55
**16**	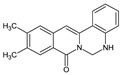	-	CH_3_	CH_3_	-	NH	78.40
**17**	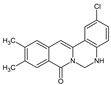	-	CH_3_	CH_3_	Cl	NH	42.86
**18**	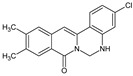	-	CH_3_	CH_3_	Cl	NH	87.96
**19**	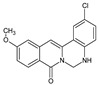	-	OCH_3_	-	Cl	NH	71.12
**20**	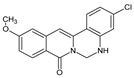	-	OCH_3_	-	Cl	NH	55.44
**21**	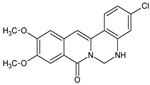	-	OCH_3_	OCH_3_	Cl	NH	50.74

## Data Availability

The data presented in this study are available in the article.
